# Supplementary material for: Factors Associated with Anthropometry Z-Scores in Exclusively Breastfed Infants Aged 0–6 Months in 10 Cities of China
Source: Nutrients. 2025 Jun 29;17(13):2163. doi: 10.3390/nu17132163 (PMC12251346; doi:10.3390/nu17132163)
Supplement: Supplementary file 1 [file nutrients-17-02163-s001.zip › nutrients-3716214-supplementary.pdf]

Table S1 Associations between infant anthropometric Z-scores and other maternal-infant related factors in univariable analyses

Variables	WAZ		LAZ		BMI Z		WLZ	
	M[P_{25},P_{75}]	*P*	M[P_{25},P_{75}]	*P*	M[P_{25},P_{75}]	*P*	M[P_{25},P_{75}]	*P*
Total	0.20 [-0.59, 1.00]		0.19 [-0.55, 0.95]		0.02 [-0.62, 1.10]		0.00 [-0.75, 0.98]	
**Demographic characteristics of mothers**								
Age (Years)		0.591		0.896		0.285		0.426
<30	0.20 [-0.57, 0.88]		0.25 [-0.55, 0.84]		-0.02 [-0.62, 0.89]		-0.14 [-0.73, 0.73]	
≥30	0.21 [-0.62, 1.21]		0.14 [-0.55, 0.96]		0.20 [-0.60, 1.25]		0.15 [-0.77, 1.00]	
Educational level		0.530		0.571		0.807		0.860
Junior high school or below	0.11 [-0.29, 0.44]		0.09 [-0.44, 0.61]		0.08 [-0.40, 0.64]		-0.12 [-0.66, 0.50]	
Vocational or senior high school	0.11 [-0.63, 0.91]		0.12 [-0.63, 0.88]		-0.04 [-0.57, 0.76]		0.00 [-0.72, 0.74]	
Junior college or above	0.25 [-0.59, 1.11]		0.25 [-0.55, 0.95]		0.03 [-0.68, 1.16]		0.00 [-0.75, 1.00]	
Monthly household income per capita (Yuan)		0.408		0.607		0.380		0.359
≤5000	0.20 [-0.62, 0.89]		0.14 [-0.55, 0.89]		-0.05 [-0.59, 0.78]		0.00 [-0.79, 0.81]	
5000~10000	0.18 [-0.61, 1.00]		0.19 [-0.50, 0.90]		0.11 [-0.69, 1.14]		0.00 [-0.82, 0.98]	
≥10000	0.50 [-0.36, 1.40]		0.43 [-0.55, 1.10]		0.34 [-0.47, 1.35]		0.25 [-0.59, 1.16]	
**Lifestyle and health status during lactation**								
GWG		0.001		0.018		0.059		0.302
Appropriate	0.20 [-0.47, 1.00]		0.21 [-0.40, 0.95]		0.07 [-0.73, 1.25]		0.00 [-0.92, 1.14]	
Inadequate	-0.17 [-0.90, 0.67]		-0.05 [-0.83, 0.48]		-0.09 [-0.87, 0.54]		-0.17 [-0.75, 0.50]	
Excessive	0.39 [-0.32, 1.32]		0.36 [-0.51, 0.95]		0.15 [-0.50, 1.14]		0.10 [-0.62, 1.00]	
Gravidity		0.072		0.195		0.043		0.156
1	0.14 [-0.66, 0.87]		0.10 [-0.60, 0.90]		-0.08 [-0.71, 0.79]		-0.10 [-0.75, 0.69]	
≥2	0.29 [-0.43, 1.21]		0.30 [-0.36, 0.95]		0.22 [-0.54, 1.30]		0.15 [-0.75, 1.03]	

Non-parametric tests, including the Kruskal-Wallis and Mann-Whitney U tests, were employed for group comparisons where appropriate.

Continued Table S1 Associations between infant anthropometric Z-scores and other maternal-infant related factors in univariable analyses

Variables	**WAZ**		**LAZ**		**BMI Z**		**WLZ**	
	$M[P_{25},P_{75}]$	*P*	$M[P_{25},P_{75}]$	*P*	$M[P_{25},P_{75}]$	*P*	$M[P_{25},P_{75}]$	*P*
Parity		0.249		0.163		0.346		0.645
1	0.19 [-0.62, 0.95]		0.14 [-0.57, 0.90]		-0.02 [-0.68, 1.02]		0.00 [-0.75, 0.86]	
≥2	0.25 [-0.44, 1.14]		0.30 [-0.35, 0.95]		0.22 [-0.55, 1.15]		0.13 [-0.77, 1.00]	
Mode of delivery		0.567		0.821		0.644		0.630
Vaginal delivery	0.20 [-0.61, 1.00]		0.14 [-0.55, 0.95]		0.04 [-0.70, 1.02]		0.00 [-0.78, 0.97]	
Cesarean section	0.20 [-0.58, 1.12]		0.25 [-0.51, 0.90]		-0.02 [-0.54, 1.18]		0.00 [-0.73, 0.93]	
Anemia		0.928		0.431		0.593		0.731
Yes	0.20 [-0.50, 1.00]		0.14 [-0.59, 0.90]		0.11 [-0.54, 0.89]		0.00 [-0.70, 0.83]	
No	0.20 [-0.62, 1.00]		0.28 [-0.35, 0.95]		-0.00 [-0.87, 1.23]		0.00 [-0.79, 1.00]	
Hypertension		0.893		0.716		0.647		0.252
Yes	0.20 [-0.59, 1.00]		0.19 [-0.55, 0.94]		0.02 [-0.63, 1.10]		0.00 [-0.75, 1.00]	
No	0.50 [-0.60, 1.06]		0.20 [-0.38, 1.45]		-0.06 [-0.48, 0.65]		-0.17 [-0.75, 0.25]	
Diabetes		0.465		0.204		0.786		0.822
Yes	0.20 [-0.57, 1.00]		0.20 [-0.55, 0.95]		0.02 [-0.63, 1.10]		0.00 [-0.76, 1.00]	
No	0.20 [-0.71, 1.00]		0.10 [-0.60, 0.50]		-0.02 [-0.54, 0.96]		0.00 [-0.74, 0.73]	
Edema		0.016		0.074		0.076		0.230
Yes	0.10 [-0.76, 0.91]		0.09 [-0.55, 0.78]		-0.06 [-0.87, 1.00]		-0.07 [-0.83, 0.84]	
No	0.39 [-0.28, 1.17]		0.36 [-0.43, 1.07]		0.17 [-0.48, 1.14]		0.08 [-0.69, 1.08]	

Non-parametric tests, including the Kruskal-Wallis and Mann-Whitney U tests, were employed for group comparisons where appropriate.

Continued Table S1 Associations between infant anthropometric Z-scores and other maternal-infant related factors in univariable analyses

Variables	WAZ		LAZ		BMI Z		WLZ	
	$M[P_{25},P_{75}]$	*P*	$M[P_{25},P_{75}]$	*P*	$M[P_{25},P_{75}]$	*P*	$M[P_{25},P_{75}]$	*P*
**Lifestyle and health status during lactation**								
Sleep time		0.030		0.191		0.211		0.762
≤8	0.17 [-0.62, 0.89]		0.14 [-0.55, 0.90]		-0.01 [-0.62, 1.01]		0.00 [-0.75, 0.86]	
>8	0.55 [-0.38, 1.41]		0.32 [-0.44, 1.27]		0.31 [-0.64, 1.24]		0.21 [-0.83, 1.18]	
Sleep quality		0.229		0.264		0.847		0.824
Poor	0.27 [-0.43, 1.14]		0.30 [-0.48, 0.95]		0.03 [-0.70, 1.14]		0.00 [-0.81, 1.00]	
Good	0.14 [-0.65, 1.00]		0.14 [-0.56, 0.91]		0.01 [-0.59, 1.04]		0.00 [-0.72, 0.90]	
Passive smoking during lactation		0.386		0.129		0.892		0.605
Yes	0.12 [-0.68, 0.88]		0.07 [-0.56, 0.41]		-0.06 [-0.46, 1.03]		0.07 [-0.48, 0.94]	
No	0.20 [-0.57, 1.00]		0.30 [-0.55, 0.95]		0.03 [-0.69, 1.12]		0.00 [-0.80, 0.96]	
Postpartum depression		0.264		0.144		0.749		0.702
Yes	0.12 [-0.60, 0.86]		0.14 [-0.55, 0.57]		0.01 [-0.63, 1.03]		0.00 [-0.75, 1.00]	
No	0.29 [-0.59, 1.14]		0.30 [-0.55, 0.95]		0.02 [-0.60, 1.12]		0.00 [-0.77, 0.87]	
Adoption of galactagogue methods		0.870		0.662		0.982		0.850
Yes	0.20 [-0.60, 1.00]		0.14 [-0.55, 0.95]		0.03 [-0.64, 1.09]		0.00 [-0.75, 1.00]	
No	0.25 [-0.54, 1.00]		0.30 [-0.35, 0.91]		-0.00 [-0.60, 1.06]		0.00 [-0.75, 0.87]	
Mastitis		0.193		0.658		0.052		0.114
Yes	0.17 [-0.61, 0.80]		0.32 [-0.55, 0.90]		-0.08 [-0.91, 0.87]		-0.17 [-0.87, 0.86]	
No	0.20 [-0.58, 1.12]		0.14 [-0.55, 0.95]		0.10 [-0.58, 1.14]		0.02 [-0.71, 1.00]	

Non-parametric tests, including the Kruskal-Wallis and Mann-Whitney U tests, were employed for group comparisons where appropriate.

Continued Table S1 Associations between infant anthropometric Z-scores and other maternal-infant related factors in univariable analyses

Variables	WAZ		LAZ		BMI Z		WLZ	
	$M[P_{25},P_{75}]$	*P*	$M[P_{25},P_{75}]$	*P*	$M[P_{25},P_{75}]$	*P*	$M[P_{25},P_{75}]$	*P*
**General information of infants**								
Sex		0.978		0.234		0.357		0.131
Male	0.20 [-0.58, 1.00]		0.33 [-0.48, 0.95]		-0.00 [-0.70, 0.96]		-0.15 [-0.87, 0.86]	
Female	0.20 [-0.60, 1.03]		0.14 [-0.57, 0.91]		0.09 [-0.59, 1.15]		0.13 [-0.54, 1.00]	
Daily Sport Time		0.515		0.310		0.657		0.062
≥30 minutes	0.18 [-0.62, 1.00]		0.16 [-0.55, 0.92]		0.04 [-0.59, 1.14]		0.22 [-0.75, 1.00]	
<30 minutes	0.20 [-0.46, 1.00]		0.19 [-0.49, 0.95]		0.01 [-0.70, 0.83]		-0.23 [-0.78, 0.73]	
Recent GI symptoms		0.043		0.485		0.067		0.078
Yes	0.13 [-0.78, 0.87]		0.09 [-0.55, 0.89]		-0.06 [-0.68, 0.76]		-0.09 [-0.83, 0.60]	
No	0.29 [-0.43, 1.14]		0.30 [-0.55, 0.95]		0.10 [-0.56, 1.16]		0.00 [-0.75, 1.10]	

Non-parametric tests, including the Kruskal-Wallis and Mann-Whitney U tests, were employed for group comparisons where appropriate.

Table S2 Model diagnostic results of the multivariate analysis

Models	WAZ	LAZ	BMI Z	WLZ
Normality of Residuals	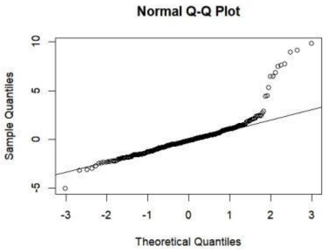	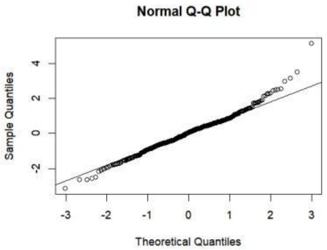	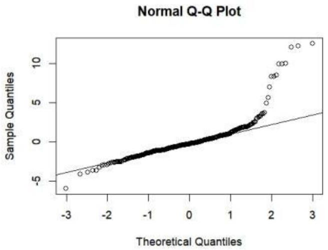	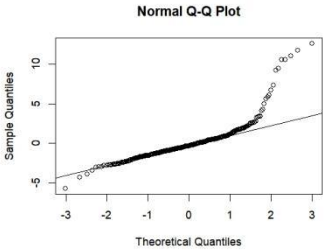
Homoscedasticity of Residuals	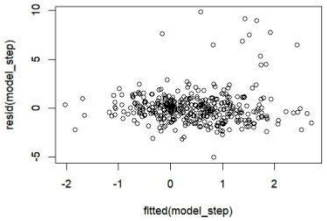	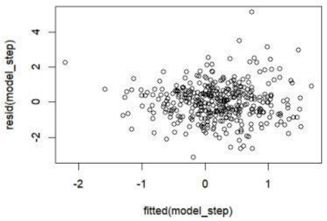	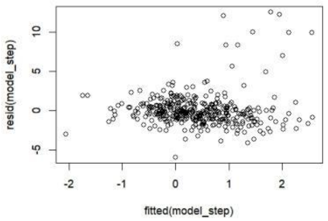	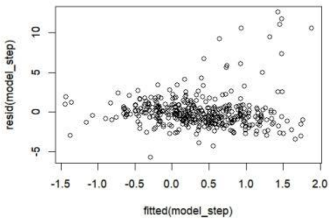
Singular	False	Ture*	False	False
Marginal R^2	0.124	0.246(R2)	0.071	0.058
Conditional R^2	0.178	0.230(Radj 2)	0.117	0.081

*: Due to singularity issues in the linear mixed-effects model, the region variable was excluded. Therefore, a general linear model was used for estimation.

Table S3 Collinearity diagnostic results of the multivariate analysis

Variable in models	VIF			
	WAZ	LAZ	BMI Z	WLZ
GWG	1.03	1.04	/	/
Pre-delivery BMI	/	/	1.02	/
Maternal weight (kg)	1.06	1.08	/	/
Paternal height (cm)	/	1.03	/	/
Maternal sleep quality	/	1.01	/	/
Maternal daily fat intake (/10g)	1.00	/	1.00	1.00
Maternal history of mastitis or ductal obstruction	/	/	1.01	1.00
Gestational weeks	/	1.07	/	/
Infant birth weight (kg)	1.02	1.14	/	/
Infant birth length (cm)	/	1.11	/	/
Infant recent GI symptoms	1.00	/	1.00	1.00

GWG: Gestational Weight Gain; BMI: Body Mass Index; GI: Gastrointestinal.
